# Hemophilus Septicemia Meningitis in an 11-Month-Old Vaccinated Infant

**DOI:** 10.7759/cureus.34991

**Published:** 2023-02-14

**Authors:** Zahraa A Alwayel, Asail S Alghamdi, Jumanah A AlmohammedSaleh, Salah Almohammed, Yameen Almatawah

**Affiliations:** 1 Medical School, King Faisal University, Al Ahsa, SAU; 2 College of Medicine, Albaha University, Al Baha, SAU; 3 Pediatric Medicine, Maternity and Children Hospital, Al Ahsa, SAU; 4 Pediatric Infectious Disease, Maternity and Children Hospital, Al Ahsa, SAU

**Keywords:** vaccination, focal seizures, brain abscesses, meningitis, haemophilus influenzae

## Abstract

*Haemophilus influenzae* is a gram-negative pleomorphic coccobacillus associated with many diseases, such as meningitis, pneumonia, septicemia, cellulitis, and otitis media. The most virulent and most common serotype is *H. influenzae* type b (Hib), which was responsible for the majority of meningitis cases until the development of vaccines that led to a decrease in its incidence worldwide. Here, we report the case of an 11-month-old female infant who was previously healthy and fully vaccinated against Hib and developed sepsis and meningitis. The patient was managed as a case of partially treated bacterial meningitis but failed to respond to a short-duration course of antibiotics and had focal seizures of the left hand. Non-contrast brain MRI revealed multiple and bilateral brain abscesses more evident on the left side. The patient was then followed up with imaging every 10-14 days to monitor the response and resolution of the brain abscesses. She was successfully treated with a full course of intravenous ceftriaxone for six weeks until imaging was clear and the brain abscesses were nearly undetected. Invasive *H. influenzae* infections are considered emerging cases, and there is a need to consider and suspect the disease even in fully vaccinated patients.

## Introduction

*Haemophilus influenzae* (*H. influenzae*) is a gram-negative coccobacillus and a common cause of various invasive and non-invasive bacterial infections that include meningitis, pneumonia, and otitis media. *H. influenzae* is divided into two main types, namely, encapsulated and non-encapsulated, which are further divided, according to the composition of the capsule polysaccharide, into six different serotypes, namely, a, b, c, d, e, and f [1,2]. *H. influenzae* type b (Hib) is the most common and virulent serotype as it accounts for most cases of *H. influenzae* invasive disease in children [3]. The incidence of Hib decreased dramatically after the initiation of routine infant vaccinations, which include the conjugate Hib vaccine that was initiated in 1998 in the Kingdom of Saudi Arabia (KSA) [4]. Although it greatly decreased the incidence of Hib infection, there was a rise in the prevalence and incidence of other serotypes and non-typable strains [5,6]. In a retrospective study of 283 patients concerning the clinical features of meningitis in general, fever was the most common presenting symptom [7]. Other symptoms depend on the age of the patient. Patients younger than six months mainly present with difficulty in feeding and bulging of fontanelle. However, patients older than two years tend to present with vomiting. Additional unspecific signs and symptoms include lethargy, seizures, petechial rash, and focal neurological signs [8]. Proper treatment is essential to prevent some of the complications that may arise from meningitis, which include hearing loss, seizures, empyema, cerebral abscess, brain edema, subdural effusion, syndrome of inadequate antidiuretic hormone, hydrocephalus, and cerebral herniation. Thus, meningitis can potentially lead to coma, hemiparesis, and even death within a matter of hours if left untreated [3].

## Case presentation

Case and physical examination

We describe the case of an 11-month-old female infant who was previously healthy and had received three doses of the Hib vaccine following the recommended vaccination schedule for children in the KSA, which includes four doses given at two, four, six, and 18 months of age. She was admitted to the Maternity and Children’s Hospital (MCH) emergency room with a fever and vomiting. On arrival, the patient was fully awake and clinically stable. The temperature was 37.5°C, the pulse rate was 130 beats per minute, and the blood pressure was 124/80 mmHg. A physical examination revealed nick stiffness but no focal neurological deficits. No rashes or lymphadenopathy were noted. Examination of the chest, heart, abdomen, ear, nose, throat, oral cavity, and pharynx did not reveal any abnormalities.

Immediate management

Empiric intravenous (IV) antibiotics, including ceftriaxone and vancomycin, were administered until culture results were available. Dexamethasone was also administered for two days to prevent any neurological damage. The patient was subsequently admitted to the pediatrics ward for further management.

Investigations

Biochemical analyses of blood showed a white blood cell (WBC) count of 11.5 × 10^3^/µL with neutrophilia. Lumbar puncture (LP) revealed a cloudy cerebrospinal fluid (CSF) with a WBC count of 610 cells/µL with predominating neutrophils, a protein count of 76.7 mg/dL, and a glucose count of 3 mg/dL. Blood and CSF were sent for gram stain and culture. The sensitivity of blood culture reached up to 50-90% and CSF culture reached more than 95% blood culture in the case of Hib meningitis. Thus, the gram stain was positive for gram-negative coccobacilli, and the culture grew *H. influenzae* without specifying its serotype due to the limited facility available at the hospital. Vancomycin was discontinued, and ceftriaxone was commenced accordingly.

Hospitalization course

**Figure 1:**
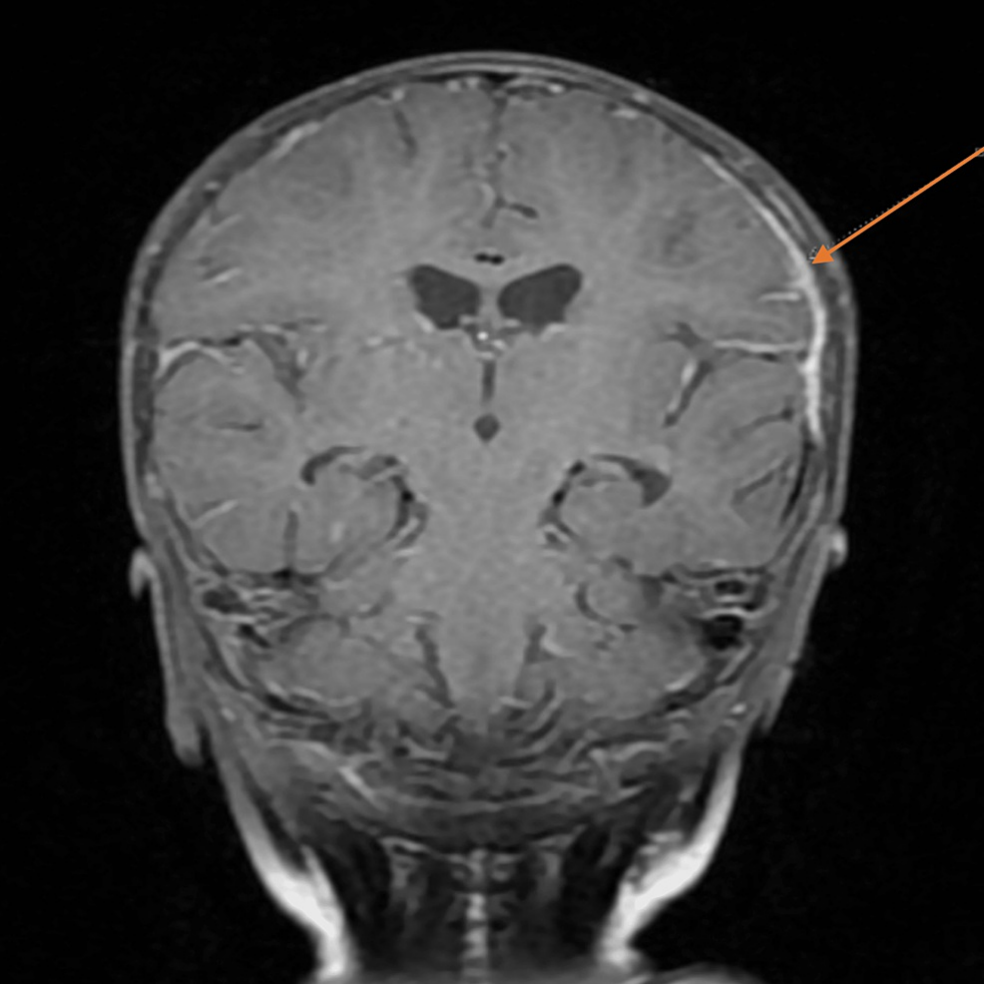
Brain MRI coronal view showing multiple bilateral small intracranial abscesses.

**Figure 2:**
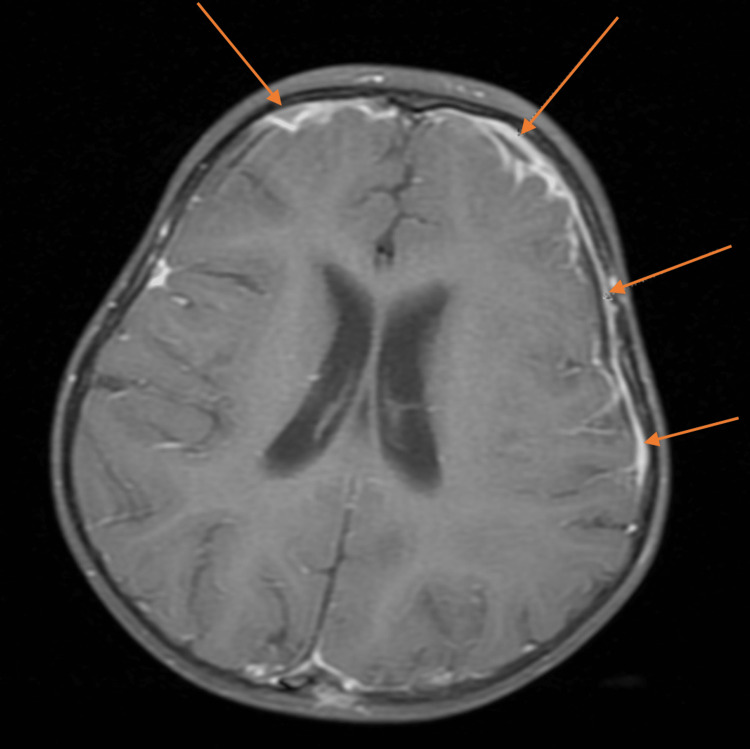
Brain MRI coronal view showing multiple bilateral small intracranial abscesses.

Five days after treatment, she developed two episodes of high-grade fever (38.6°C and 38.3°C) and multiple focal seizures of the left hand. Both electroencephalogram (EEG) and contrast-enhanced MRI of the brain were ordered. EEG, performed using the standard 10-20 system, was normal. The brain MRI revealed multiple bilateral small intracranial abscesses, which were more dominant on the left side (Figures 1, 2). A neurologist was consulted for further management, and medical therapy was considered to be more appropriate for the patient as she had no indications for surgical removal. Ceftriaxone was continued for six weeks without any additional episodes of neck stiffness or seizures. Brain MRI was repeated every 10-14 days during this period to monitor the resolution of abscesses with treatment.

Follow-up

**Figure 3:**
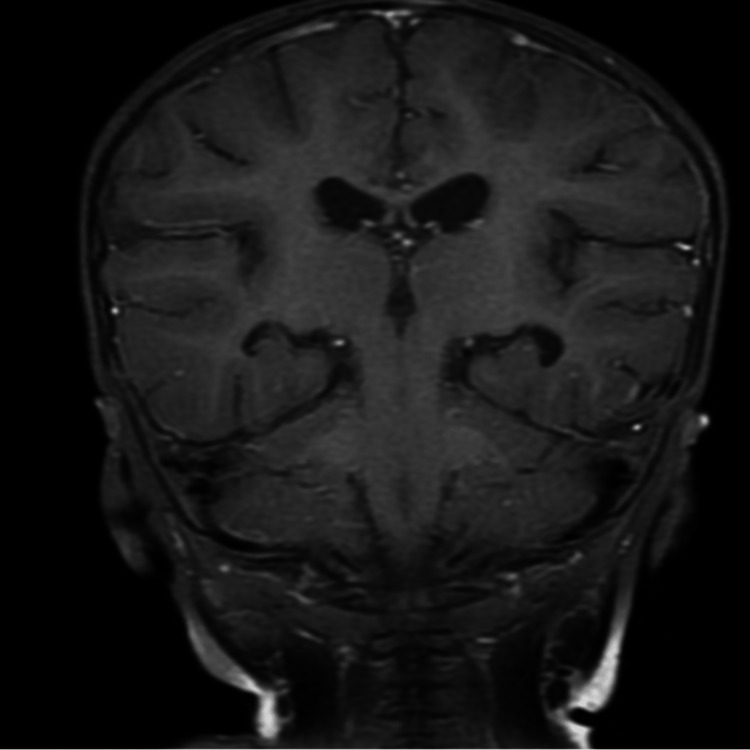
Follow-up MRI coronal view showing nearly complete resolution of the brain abscesses six weeks after treatment.

**Figure 4:**
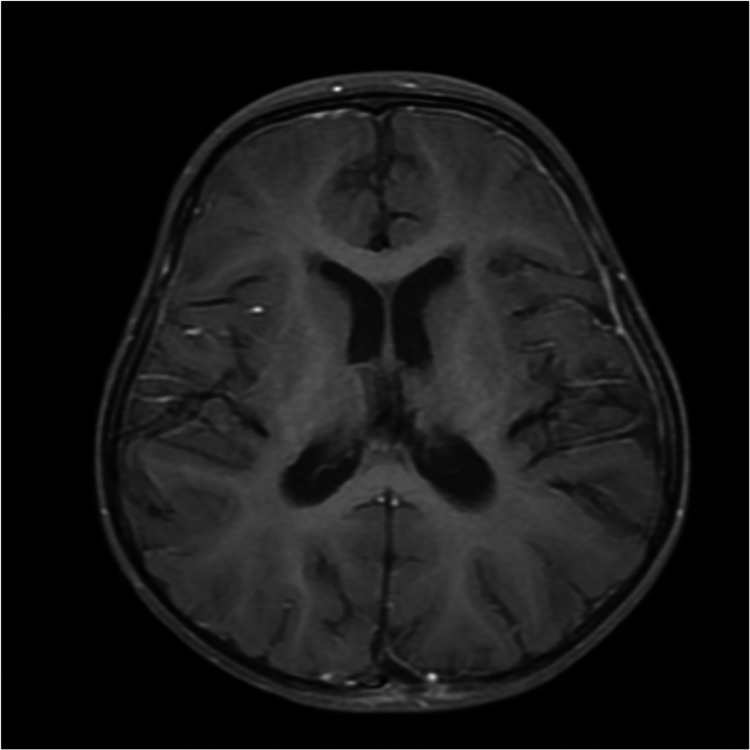
Follow-up brain MRI axial view showing nearly complete resolution of the brain abscesses six weeks after treatment.

Six weeks after treatment, the brain abscesses were almost completely resolved, and the patient successfully recovered without any neurological sequelae (Figures 3, 4). The patient was then followed up in the hospital outpatient department for hearing and visual assessment, both of which were normal.

## Discussion

Our patient was fully immunized and had no known risk factors. Despite the fact that *H. influenzae* meningitis has been eliminated since the beginning of vaccination, it may occur due to vaccination failure, which should be taken into account when treating patients with suspected meningitis.

Following vaccinations, a few cases of pediatric meningitis were attributed to Hib, and to a lesser extent, other strains. We found a recent case report of an invasive disease caused by Hib with an atypical presentation that occurred in a partially vaccinated two-year-old boy in the United Arab Emirates in 2021. This case demonstrated how important it is to consider Hib in the differentials of bacterial meningitis even when the symptoms are unspecific and resemble mild viral illnesses of the upper and lower respiratory and gastrointestinal tracts. This is because bacterial meningitis can be serious and possibly fatal if not diagnosed and treated early.

To our knowledge, this is the third case reported in the KSA regarding an invasive disease caused by *H. influenzae* despite receiving the Hib vaccine according to the appropriate age; the first two cases were reported in a case series from Riyadh in 2016. The first patient was a medically free 10-month-old boy who had received two doses of the Hib vaccine and presented with a two-day duration of fever and vomiting. The patient was discharged in good health after receiving a 10-day course of ceftriaxone and vancomycin, which was discontinued after receiving the culture that revealed type A *H. influenzae* (Hia). The second patient was a three-month-old boy who received one dose of the Hib vaccine at two months of age and presented with fever and irritability for five days. The patient was diagnosed with meningitis and sepsis and was further managed with vancomycin, ampicillin, and ceftriaxone based on the culture that was positive for Hia [9].

Another case of meningitis caused by Hia was reported from Oman in 2015 in a previously healthy 17-month-old girl who was admitted due to a one-day history of fever, lethargy, frequent vomiting, and a refusal of food for six to eight hours before admission. She had all of her vaccinations up to date. The patient had a protracted clinical course, characterized by continued photophobia, intermittent fever (38-39°C), and subdural effusion. Her condition was managed by 10 days of IV ceftriaxone, changed to IV ampicillin for two weeks, and then she was discharged without any complications [10].

There have also been reports of cases in Brazil, including one involving a five-month-old girl who was extremely irritable and had a high-grade fever for three days, and her parents reported “jet-like vomiting.” The patient experienced another episode of jet-like vomiting and one tonic-clonic convulsion during her 65 days of hospitalization. The case was managed with IV ceftriaxone, IV dexamethasone, and sodium dipyrone for fever [11].

Although brain abscess is a very rare complication, it should be considered in all patients with Hib meningitis if it does not improve or deteriorates with complications despite treatment. A case report involving a seven-month-old patient documented Hib meningitis complicated with brain abscess formation. The patient was fortunately successfully managed with needle aspiration and four weeks of antibiotic therapy with ampicillin and chloramphenicol [12]. Our patient was not only fully vaccinated but had developed multiple brain abscesses as was evident on brain MRI which mandated a longer course of antibiotic therapy of six weeks until she improved and brain imaging was clear.

## Conclusions

Advanced brain imaging is recommended for all patients with bacterial meningitis with an emphasis on patients not improving or deteriorating after initial improvement for the possibility of developing intracranial abscesses, particularly in cases of *H. influenzae* even in young vaccinated individuals. Following up patients with brain imaging along with monitoring clinical improvement is very important as it has been considered to guide the duration of antibiotics. We have high hopes that our case report will motivate more research to be conducted on the occurrence of meningitis and other invasive illnesses caused by *H. influenzae*, as well as raise suspicion of *H. influenzae* meningitis among doctors, even in completely vaccinated patients.

## References

[1] Almeida AF, Trindade E, B Vitor A, Tavares M (2017). Haemophilus influenzae type b meningitis in a vaccinated and immunocompetent child. J Infect Public Health.

[2] Campos J, Hernando M, Román F (2004). Analysis of invasive Haemophilus influenzae infections after extensive vaccination against H. influenzae type b. J Clin Microbiol.

[3] Khattak ZE, Anjum F (2023). Haemophilus Influenzae. StatPearls - NCBI Bookshelf. Haemophilus Influenzae - StatPearls - NCBI Bookshelf [Internet].

[4] Almuneef M, Alshaalan M, Memish Z, Alalola S (2001). Bacterial meningitis in Saudi Arabia: the impact of Haemophilus influenzae type b vaccination. J Chemother.

[5] Bruce MG, Zulz T, DeByle C (2013). Haemophilus influenzae serotype a invasive disease, Alaska, USA, 1983-2011. Emerg Infect Dis.

[6] Urwin G, Krohn JA, Robinson KD, Wenger JD, Farley MM (1996). Invasive disease due to Haemophilus influenzae serotype f: clinical and epidemiologic characteristics in the H. influenzae serotype b vaccine era. Clin Infect Dis.

[7] Türel Ö, Yıldırım C, Yılmaz Y, Külekçi S, Akdaş F, Bakır M (2013). Clinical characteristics and prognostic factors in childhood bacterial meningitis: a multicenter study. Balkan Med J.

[8] Qureshi MA, Asad I, Chaudhary A, Abuhammour W (2021). Beta-lactamase-negative ampicillin-resistant Haemophilus influenzae type b meningitis in partially immunized immunocompetent child: a case report. J Med Case Rep.

[9] Roaa Z, Abdulsalam A, Shahid G, Kamaldeen B, Tariq AF (2016). Pediatric invasive disease due to Haemophilus influenzae serogroup A in Riyadh, Saudi Arabia: case series. J Infect Dev Ctries.

[10] Sawardekar KP (2017). Haemophilus influenzae type a meningitis in immunocompetent child, Oman, 2015. Emerg Infect Dis.

[11] Falleiros de Pádua RA, Lima Scodro RB de, Ghiraldi LD, Dias Siqueira VL, Yamashita YK, Helbel C, Rosilene FC (2009). Haemophilus influenzae serotype a meningitis. Ann Clin Lab Sci.

[12] Feldman WE, Schwartz J (1983). Haemophilus influenzae type b brain abscess complicating meningitis: case report. Pediatrics.

